# Assessment of the Impact of Nutritional Intervention with the Probiotic *Lactiplantibacillus plantarum 299v* on Nutritional Status and Quality of Life of Hashimoto’s Thyroiditis Patients—A Randomized Double-Blind Study Protocol

**DOI:** 10.3390/jpm13121659

**Published:** 2023-11-28

**Authors:** Karolina Osowiecka, Damian Skrypnik, Joanna Myszkowska-Ryciak

**Affiliations:** 1 Doctoral School, Warsaw University of Life Sciences (WULS), 02-787 Warsaw, Poland; 2 Department of Dietetics, Institute of Human Nutrition Sciences, Warsaw University of Life Sciences (WULS), 02-776 Warsaw, Poland; 3 Department of Treatment of Obesity, Metabolic Disorders and Clinical Dietetics, Poznan University of Medical Sciences, 60-569 Poznan, Poland

**Keywords:** Hashimoto’s thyroiditis, nutrition, probiotic, intervention, nutritional status, quality of life, female

## Abstract

The current treatment for the autoimmune disease of hypothyroidism (AIDH) is based on pharmacotherapy with levothyroxine. A non-pharmacological supplementary element of therapy could be the implementation of an individualized balanced diet and probiotics. *Lactiplantibacillus plantarum 299v* (*Lp299v*), with its anti-inflammatory effects, may also support the therapy. However, the number of studies on personalized dietary interventions with probiotics in AIDH is limited, and no clear conclusions can be drawn from the results so far. Therefore, this trial will analyze the effect of *Lp299v* supplementation in conjunction with nutrition education on the quality of life and nutritional status of patients with Hashimoto’s. Methods: This double-blind, 12-week intervention study will include 100 female patients with AIDH. They will be divided into two groups: (1) individual personalized nutrition education + *Lp299v* and (2) individual personalized nutrition education + placebo. Before and after the education intervention, selected elements in the diet, eating behavior, quality of life, nutritional status (anthropometric parameters, body composition), blood pressure, and anti-TPO (antibodies against thyroid peroxidase) titer will be assessed. Hypothesis: It is expected that this study will provide deeper knowledge on the validity of using proper nutritional principles and *Lp299v* in AIDH. Specifically, the impact on the subjective assessment of the quality of life, selected elements in the diet, and the state of nutrition and health will be assessed.

## 1. Introduction

The autoimmune disease of hypothyroidism (AIDH, Hashimoto’s thyroiditis) affects an increasing number of people, particularly women [1]. In the course of this disease, many symptoms and health consequences arise that worsen the quality of life [2]. Hypothyroidism worsens the motility of the gastrointestinal tract, which may lead to disorders such as dyspepsia, IBS (irritable bowel syndrome), or SIBO (Small Intestinal Bacterial Overgrowth) [3,4]. Hypothyroidism may also increase the risk of obesity [5], which is associated with a positive anti-TPO (antibodies directed against thyroid peroxidase) titer [6]. Patients with AIDH are more likely to have excess body mass compared to those without the disease [5], which increases the risk of hypertension—the most important risk factor for ischemic heart disease, stroke, other cardiovascular diseases, chronic kidney disease, and dementia [7]. On the other hand, subclinical hypothyroidism may also occur during the course of AIHD, increasing the risk of hypertension, especially in middle-aged women, although the mechanism is not fully understood [8]. The treatment for AIHD mainly involves the patient taking levothyroxine for life [2].

The medical treatment of AIDH can be supplemented by a balanced diet containing important anti-inflammatory nutrients. Selenium and vitamin D reduce the anti-TPO and anti-TG antibodies (antibodies directed against thyroglobulin) characteristic of AIDH [9,10,11,12,13,14]. In addition, selenium and iodine are key elements in thyroid hormone synthesis, and their deficiency can lead to hypothyroidism [15,16,17]. Zinc, copper, and iron also affect the production of thyroid hormones [18]. A low iron status results in less efficient use of iodine and thyroid hormone synthesis, leading to hypothyroidism [17,19]. In addition, iron-deficiency anemia is correlated with a higher frequency of anti-TPO antibodies [20]. Recent reports indicate that the higher the serum magnesium level, the lower the titer of anti-TG antibodies [21]. Literature reviews have mainly focused on the impact of nutrients on Hashimoto’s thyroiditis, with few articles on the type of diet that would be most beneficial [22]. Most often, patients are recommended an anti-inflammatory, balanced diet, with the Mediterranean diet being the most recognizable example. Ruggeri et al. [23] observed that adhering to the Mediterranean diet was associated with a lower odds ratio of developing thyroid autoimmunity. The anti-inflammatory diet has also found applications in systemic lupus erythematosus [24], multiple sclerosis [25], psoriasis [26], and rheumatoid arthritis [27].

A diet that is especially rich in fermented food products and drinks containing probiotic bacteria has been associated with health benefits, such as a reduced risk of high blood pressure [28] and obesity [29]. There is growing evidence of the beneficial effects of probiotics, not only on the intestinal microbiota but also on overall health, including immune health [30]. Unfortunately, the number of studies on the impact of probiotic therapy on Hashimoto’s thyroiditis is unsatisfactory. In a blinded study conducted by Talebi et al., a synbiotic blend was used, consisting of four strains of the genus *Lactobacillus,* two strains of the genus *Bifidobacterium*, *Sreptococcus thermophilus*, and fructooligosaccharide. This blend showed no significant effect on anti-TPO [31]. Another study examined the effects of a synbiotic (500 mg of 10^9^ CFU/g probiotics plus fructooligosaccharide) on adult hypothyroid patients [32]. Although a 10-week supplementation had no beneficial effects on serum thyroid-stimulating hormones, it did result in improved blood pressure and quality of life among patients with hypothyroidism [32]. Hashimoto’s thyroiditis is characterized by an increased inflammatory burden, accompanied by increased red cell distribution width. The latter increases in iron deficiency anemia but is also associated with conditions characterized by overt or subclinical inflammation [33]. On the other hand, nutritional status and indexes, such as the prognostic nutritional index (PNI), are also associated with inflammatory conditions, such as infections [34] and diabetic microvascular complications [35]. Thus, the effects of altering nutritional status via probiotic bacteria in Hashimoto’s thyroiditis are worthy of further study.

Based on a wide search of the literature, it was found that the data on dietary interventions and probiotic therapy for AIDH are unsatisfactory. Furthermore, it is difficult to determine the efficacy of effective probiotic therapy in AIDH, especially since the results of studies on specific strains cannot be extrapolated to other strains of the same species that have not been tested in patients with AIDH. In addition, no studies were found on interventions consisting of rational nutrition in combination with probiotic therapy (*Lactiplantibacillus plantarum 299v*) in individuals with AIDH aimed at improving their diet, nutritional status, and quality of life. Therefore, this study will provide further data on this relatively unexplored area of research.

## 2. Study Aim and Hypothesis

The primary objective of the trial is to assess the effect of a dietary intervention with probiotic *Lp299v* supplementation on body weight and quality of life in female AIDH patients.

The secondary objective of the trial is to evaluate the effectiveness of individual personalized nutritional counseling on several key parameters among patients diagnosed with Hashimoto’s thyroiditis. The specific focus areas for assessment include food selection and diet quality, body composition, serum anti-TPO, and blood pressure.

The research hypotheses are as follows:

- Individual personalized nutrition education aimed at improving food selection and diet quality may enhance nutritional status, anti-TPO parameters in the blood, and the overall quality of life in AIDH patients.
- The *Lp299v* strain may augment the impact of the individual nutritional intervention, particularly in terms of improving the quality of life, body weight status, and anti-TPO parameters in the blood.

## 3. Materials and Methods

### 3.1. Study Design and Ethical Approval

We plan a 12-week single-center, double-blind dietary intervention in which AIDH patients will be randomly allocated into two groups: experimental and control. Participants in the experimental group will receive individual personalized nutrition education along with the probiotic *Lactiplantibacillus plantarum 299v*, while in the control group, *Lp299v* will be replaced by a placebo. The form of the administered supplement (capsule) will be identical in both cases; neither the researcher nor the patient will have knowledge of its contents. The detailed schedule of the trial is presented in the following section. All visits for measurements and data collection will take place at the Dietetics Counselling Center, Department of Dietetics, Faculty of Human Nutrition, Warsaw University of Life Sciences. All tests performed during the intervention will be provided free of charge to the participants, who will not receive any financial benefits or cost reimbursements for participating in the study.

The study protocol was registered with and approved by the Ethics Committee for Research with Human Participation at the Institute of Human Nutrition Sciences, Warsaw University of Life Sciences, in compliance with the principles of ethics (Resolution No. 22/2021 and No. 21/2022). The study will adhere to the Declaration of Helsinki, and all participants will provide informed consent. Each participant will be presented with a detailed description, and any questions or concerns will be addressed. Participants will be informed that they may withdraw at any stage from the study without providing a reason. It is important to note that side effects of *Lactiplantibacillus plantarum 299v* are rare and do not pose a threat to the patient. To ensure participant retention, direct contact with the researcher will be available throughout the study period.

### 3.2. Selection of Participants

The selection of the sample for the study will be purposive, taking into account the inclusion and exclusion criteria. Recruitment for the study will be carried out using the snowball method, mainly via social networking sites (i.e., internet websites associated with Hashimoto’s patients). Additionally, collaboration with medical facilities and pharmacies will be established to reach a broader pool of potential participants. The CAWI (Computer-Assisted Web Interview) and PAPI (Paper and Pen Personal Interview) methods will be used for collecting data.

The inclusion criteria will be a medical diagnosis of Hashimoto’s disease based on anti-TPO, anti-TG, or an ultrasound image characteristic of AIDH; thyroid function status (euthyroid, hypothyroidism); female; age in the range of 18-64 years; normal body weight (body mass index BMI 18.50–24.99 kg/m^2^) or excessive body weight (BMI ≥ 25.00 kg/m^2^); and/or low-quality diet (low Pro-Healthy Diet Index (pHDI-10)) [36]. Women with contraindications to a body composition analysis performed using the electrical bioimpedance method (i.e., having a pacemaker) will also be included. However, for these patients, the body composition analysis stage will be skipped.

The exclusion criteria will be thyroid diseases other than AIDH, pregnancy, lactation, diagnosed cancer, and lack of consent to participate in the study. The individual must not take *Lp299v* or medications for weight loss or be diagnosed with celiac disease, Duhring’s disease, or a gluten allergy. The minimum number of subjects (47 for each group) was calculated based on an expected 50% improvement in the overall quality of life after 12 weeks of the intervention in the experimental group, with an assumed 5% significance level and 80% power. The baseline sample size of 100 patients was established to account for potential participant withdrawals.

### 3.3. Randomization and Blinding

Participants will be randomly allocated, according to a generated randomization list, to the experimental group (approximately *n* = 50) or the control group (approximately *n* = 50). The randomization list will be generated by a researcher who is not involved in the experiment. The study is designed as double-blinded, meaning that neither the participant nor the intervention conductor will be aware of which group is the experimental group (receiving *Lp299v*) and which is the control group (receiving placebo) until the end of the experiment. Each patient will be assigned a unique code number, which will be used to label all results to maintain blinding.

### 3.4. Course of Intervention

**Figure 1:**
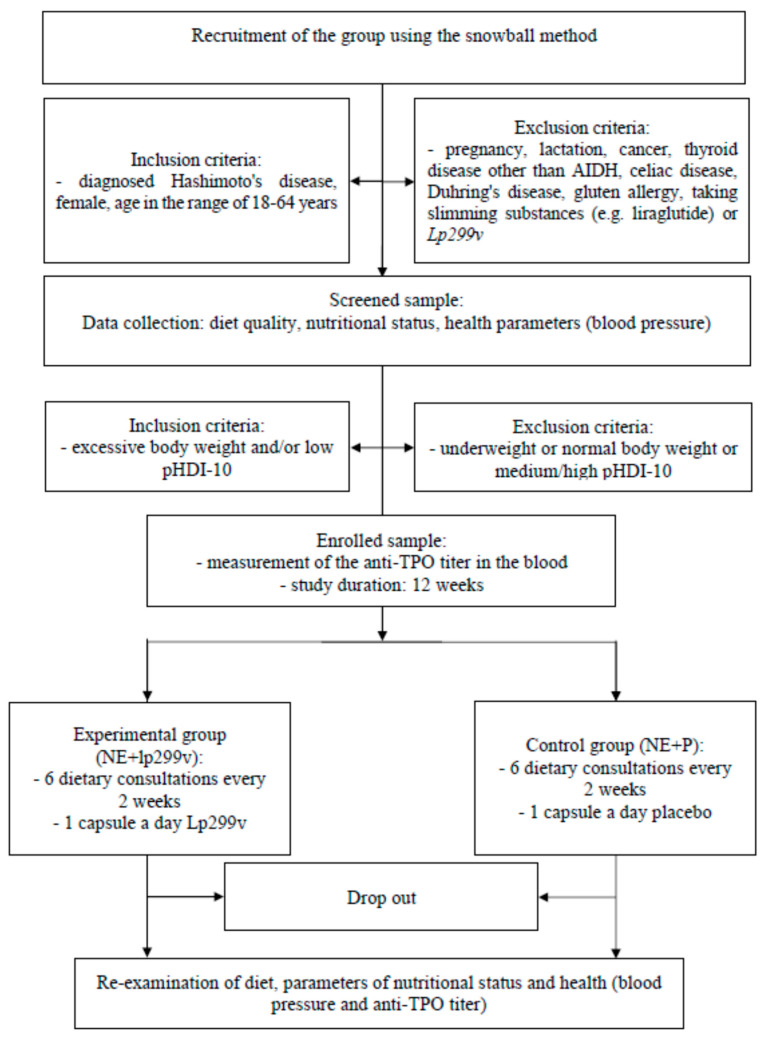
The study diagram. AIDH—autoimmune disease of hypothyroidism; pHDI-10—Pro-Healthy Diet Index 10; NE + *Lp299v*—nutrition education (1 dietary consultation every 2 weeks) + *Lactiplantibacillus plantarum 299v*; NE + P—nutrition education (1 dietary consultation every 2 weeks) + placebo; *Lp299v*—*Lactiplantibacillus plantaum299v*.

The 12-week intervention will involve individual nutrition education for patients with Hashimoto’s thyroiditis conducted in two groups. One group will additionally receive the *Lp299v* strain (NE + *Lp299v* group), and the other will receive a placebo (NE + P group). Before the start of education, an assessment of dietary habits will be performed using a three-day food record questionnaire. Additionally, validated tools will be used to assess (1) the dietary habits and nutritional beliefs (FFQ-6 and KomPAN^®^ questionnaire [36,37]); (2) the quality of life with thyroid diseases (ThyPROpl) [38]; (3) physical activity level (Polish version of IPAQ questionnaire) [39]. Anthropometric measurements (body weight and height, waist and hip circumferences), body composition, peripheral arterial pressure, and anti-TPO blood concentration measurements will also be performed. All parameters will be measured twice: at baseline and after 12 weeks of the intervention to evaluate changes and (if possible) compare the measurements to reference values. A diagram of the study is presented below (Figure 1).

### 3.5. Nutrient Intake and Diet Quality

The energy and nutrient intake will be assessed using the three-day food record method recommended by The Polish Society of Dietetics and the National Consultant of Family Medicine [40]. Participants will be asked to record everything that they eat and drink in household measures or grams on three non-consecutive days (two typical and one non-typical). Before completing the food record, patients will be instructed on how to properly fill out the questionnaire and will receive an example of a completed form to illustrate the task. After returning the completed food record, the average energy and nutrient intake will be calculated for each patient using a special computer application, “DietetykPro^®^” (Poland), with the Polish nutrient database for food products and dishes [41]. In the absence of a recorded product, the United States Department of Agriculture (USDA) nutrient database [42] will be utilized, or the most similar product will be chosen to calculate the nutrient content. Diet adequacy will be assessed by comparing the average intake of nutrients (macronutrients, minerals, and vitamins) with the Polish nutritional standards and recommendations [43]. Energy intake will be compared to the individual’s total energy needs, estimated by multiplying the individual’s basal metabolic rate (calculated with Mifflin St Jeor equations [44]) by the physical activity factor (physical activity level (PAL): 1.4 to 1.69—sedentary or light-activity lifestyle; 1.7 to 1.99 active or moderately active lifestyle; 2.00–2.40 highly physically active lifestyle) of the patient [45].

To assess the consumption frequency of selected food products and eating habits, the Polish-validated Food Frequency Questionnaire (FFQ-6) will be used. The questionnaire covers 62 different food products. Participants must choose one of the six categories indicating the frequency of food consumption over the past 12 months: (1) never or almost never, (2) once a month or less, (3) several times a month, (4) several times a week, (5) daily, (6) several times a day. The questionnaire also includes socio-demographic questions necessary to characterize the group [37].

To assess the overall quality of the diet, the following Polish-validated tools will be used: the Pro-Health Diet Index (pHDI-10) and the non-Healthy-Diet Index (nHDI-14) [36]. The first one includes ten food items: whole-grain bread/rolls, coarse-grounded groats, milk, fermented milk beverages, fresh cheese curd products, white meat, fish, legume-based foods, fruit, and vegetables. The latter includes fourteen food items: white bread and bakery products, white rice and fine-ground groats, fast foods, fried foods, butter, lard, cheese, cured meat/smoked sausages/hot dogs, red meat, sweets, tinned meats, sweetened beverages, energy drinks, and alcohol. Based on the answers provided by each individual, the total points are recalculated to a range of 0%–100% according to the questionnaire’s manual. The index value reflects the intensity of beneficial or harmful characteristics for health. For example, pHDI-10 scores ranging from 0 to 33 indicate low diet quality [36].

### 3.6. Selected Lifestyle Factors

The participants’ levels of physical activity will be assessed using the International Physical Activity Questionnaire (IPAQ), validated by Biernat et al. [39]. The Polish version quantifies physical activity in metabolic equivalent of task (MET)-min/week units, which allows the respondents to be easily classified into one of three categories of activity: insufficient (below 600), sufficient (600–3000), or high (above 3000 MET-min/week). The questionnaire comprises two parts: The first includes six brief questions (with Yes/No responses), designed to verify whether the preceding seven days were representative of the participant’s typical level of physical activity. In the second part, participants answer seven questions related to physical activities at both intense and moderate levels, as well as all walking and sitting [39].

Self-reported data on health status, sleep duration, and smoking status will be collected using the KomPAN questionnaire [36]. Additionally, all participants will be queried about ongoing medical care, medications, and dietary supplements and herbs, along with an assessment of their stress levels.

### 3.7. Quality of Life

The quality of life of the participants will be assessed using the Quality of Life Questionnaire for Patients with Thyroid Diseases (ThyPROpl).

ThyPROpl is a thyroid-specific questionnaire validated for the Polish population by Sawicka-Gutaj et al. [38]. The questionnaire comprises 85 questions grouped into 13 scales measuring aspects of quality of life relevant to thyroid patients. The 13-point scale includes questions on (1) symptoms, (2) feeling tired, (3) energy, (4) memory and concentration, (5) nervousness and tension, (6) mental well-being, (7) problems with coping or mood swings, (8) relationships with other people, (9) daily activities, (10) sex life, (11) the impact of the disease or its treatment on appearance, (12) the intensity of the impact of the disease on the subject in general. All quality-of-life questions will cover the last 4 weeks prior to the survey. The respondents must choose 1 of 5 answer categories: (1) not at all, (2) a little, (3) average, (4) quite a lot, (5) very much. In the question regarding difficulties in performing duties at work, the authors added an additional category—“I do not work” [38]. The score ranges from 0 to 100, with higher scores indicating a poorer quality of life.

### 3.8. Nutritional Status

Body weight will be measured with the subject in a standing position with an electronic digital scale. The examined person will be weighed in her underwear without shoes, and the scores will be read out with an accuracy of 100 g [46].

Body height will be measured with a medical scale with a built-in height gauge with the subject in the standing anthropometric position (lower limbs straightened, feet set parallel to each other, upper limbs straightened, hanging loosely along the trunk, head set in the eye–ear plane). The individual will have her shoes and socks off, as well as hair ornaments. The measurement will be taken from the top of the head to the base on which the person is standing. Measurements will be read out with an accuracy of 1 mm [46].

Waist circumference will be measured at the midpoint between the lower margin of the least palpable rib and the top of the iliac crest, with the subject in a standing position with body weight evenly distributed between both feet, after emptying the bladder and with relaxed muscles, with an accuracy of 1 mm [47]. Hip circumference will be measured at the widest part of the buttocks, with the subject in a standing position with body weight evenly distributed between both feet, after emptying the bladder and with relaxed muscles, with an accuracy of 1 mm [47].

Based on anthropometric data, body mass index (BMI = weight (kg)/(height (m))^2^) [48], waist-to-hip ratio (WHR = waist circumference (cm)/hip circumference (cm)) [47], and waist-to-height ratio (WHtR = waist circumference (cm)/height (cm)) will be calculated [49]. Overweight will be defined as a BMI between 25.00 and 29.99 kg/m^2^, while obesity will be defined as a BMI ≥ 30.00 kg/m^2^. Central obesity will be defined as WHR > 0.8 [47] and/or WHtR ≥ 0.5 [50].

Body composition will be analyzed with the bioelectrical impedance (BIA) method using the body composition analyzer (ACCUNIQ BC-720). The measurement will be conducted with the subject in a standing position. The individual will stand barefoot in her underwear, and the measurement will be taken at least 4 h after a meal and at least 12 h after intense physical activity. The tested person will avoid consuming caffeine-containing beverages (e.g., coffee, energy drinks), use the toilet before the measurement (defecation and urination), and remove jewelry and any metal elements (e.g., a belt). The body composition analyzer will provide data on muscle, total fat, visceral tissue, and water content in the body.

### 3.9. Blood Pressure

At least 30 min before the measurement, the patient should refrain from consuming coffee, smoking cigarettes, and taking other stimulants [51]. Peripheral pressure will be examined with the subject in a sitting position, after a minimum of 5 min of rest, with the ACCUNIQ BC-250. The patient will be in a sitting position with her back supported in a quiet room, with thermal comfort maintained. The arm on which the measurement is made should be free of constricting clothing, bent at the elbow, relaxed, and supported at the level of the patient’s heart [51].

### 3.10. Anti-TPO Titer Concentration

The anti-TPO titer will be determined in venous blood taken from a peripheral vein of the forearm by qualified personnel from the medical laboratory. The concentration will be determined by immunochemistry on the “Alinity i” laboratory instrument and compared to a reference value of <5.61 IU/mL.

The factors influencing the anti-TPO result are anti-streptavidin and anti-ruthenium antibodies. Therefore, the measurement will be omitted for these patients. Biotin is also a factor that interferes with the anti-TPO titer measurement. Therefore, patients taking biotin supplementation will be advised to take a minimal break the day before the examination [52].

### 3.11. Nutrition Education

Nutrition education is planned for 12 weeks, with 6 individual meetings spaced about 2 weeks apart. There will be a total of 6 h of education in the entire program. The form of meetings will be online or in person, depending on the patient’s preferences. At the beginning of the education intervention, the patient will be presented with an interpretation of her eating habits, the nutritional value of her diet, and her nutritional status. A personalized goal (e.g., weight loss in the case of a patient with obesity) that the patient should achieve will also be discussed and set. During the education intervention, the following issues, among others, will be discussed with all participants: nutritional recommendations and essential nutrients (role, sources in food) in Hashimoto’s thyroiditis; factors affecting iron bioavailability; glycemic index and glycemic load—application and importance; weight reduction assumptions; snacking—causes and tips on how to minimize the need to reach for snacks; mindful eating; “Healthy eating plate”—nutritional recommendations for the Polish population presenting the correct proportions of individual product groups in a daily diet [53]; interactions between levothyroxine and food; the importance of microbiota in HT; *Lactobacillus plantarum 299v*—impact on human health based on scientific research (mainly on the immune system, iron, intestinal symptoms, blood pressure); and myths and facts about nutrition in HT. In addition to the above-mentioned topics, participants will be able to receive answers to individual questions/concerns.

All educational materials will be prepared based on the scientific literature and standards by The Polish Society of Dietetics and the National Public Institute—National Institute of Hygiene. Each participant will receive educational materials and a sample menu depending on energy needs to illustrate how meals and portions should look. Throughout the entire study period, participants will have the possibility of consulting with a dietitian, who will also support the introduction of changes in eating habits depending on individual personalized needs.

### 3.12. Lactiplantibacillus plantarum 299v

The experimental group will take 1 × 10^10^ CFU (colony-forming units) of *Lactiplantibacillus plantarum 299v* contained in Sanprobi IBS^®^ once a day with a meal. The carrier will be potato starch and magnesium salts of fatty acids (capsule shell: hydroxypropyl methylcellulose). The control group will take a capsule with a placebo, i.e., granulated potato starch and magnesium stearate (capsule shell: hydroxypropyl methylcellulose), also once a day with a meal. At each educational meeting, participants will be asked whether they are taking the given capsules and whether they have experienced any side effects. After the intervention, participants will be asked about any missed doses of capsules. Throughout the study period, both the probiotics and the placebo will be stored in the refrigerator, following the manufacturer’s recommendations. *Lp299v,* contained in the preparation “Sanprobi IBS^®^”, is registered with the Chief Sanitary Inspector. In addition, the preparation received a positive evaluation from the Institute Of Monument Children’s Health Center (IPCZD) No. 5/DJW/2020 and the Quality Institute of the Jagiellonian Center of Innovation, which states that SANPROBI IBS ^®^ has good probiotic properties and is safe for the consumer. The manufacturer declares that the amount of the probiotic strain in CFU will remain consistent throughout the product’s shelf life.

### 3.13. Statistical Analyses

All statistical analyses will be conducted using Statistica 13.1 PL (StatSoft Inc., Tulsa, OK, USA; StatSoft, Krakow, Poland). Categorical variables will be presented as a sample percentage (%). Continuous variables will be presented as means with 95% confidence intervals (95%CIs) for variables with a normal distribution or medians with an interquartile range (IQR) for variables with a non-normal distribution. To assess the impact of the nutritional intervention with probiotic supplementation and verify the differences between the experimental and control groups, multivariate analysis of variance and two-tailed tests will be applied. The normality of the variable distribution will be assessed using the Shapiro–Wilk test before statistical analysis. For continuous variables, e.g., diet quality scores, scores of quality of life, markers of adiposity, metabolic parameters, and changes after 12 weeks of education relative to baseline, will be assessed using a *t*-test for dependent samples for normally distributed variables or a Mann–Whitney test for variables without a normal distribution. Differences in categorical variables will be examined using the Pearson chi-squared test. *p* < 0.05 will be considered statistically significant.

## 4. Discussion

To the best of our knowledge, this is the first study assessing the impact of diet and probiotic supplementation on anthropometric, body composition, and biochemical parameters (anti-TPO), as well as the quality of life, among women with Hashimoto’s disease. Existing evidence-based dietary recommendations for Hashimoto’s disease are deemed insufficient, as indicated by a systematic review [22]. Enhancing a patient’s ability to adopt beneficial nutritional modifications in the diet can be achieved through targeted nutrition and dietary education. A systematic review by Silva-Santos et al. demonstrated that education successively reduced salt intake in adults [54]. In the context of cancer, nutrition education with dietary counseling has reduced malnutrition in patients undergoing radiotherapy [55]. It has also led to a significant improvement in the quality of life during adjuvant chemotherapy in patients with breast cancer [56]. Education also played a role in minimizing muscle loss in hemodialysis patients [57].

The duration of education is crucial in achieving results. According to Murimi et al.’s systematic review, effective education should last more than 5 months [58]. There are also measurable benefits from the implementation of 12-week education [59,60,61]. In order for dietary education to be more effective, it should be conducted by a dietitian, especially through individual consultations [59,62]. The number of studies investigating the use of probiotics in AIDH is also limited. There is a great body of literature regarding the relationship between the microbiota and AIDH [63,64,65,66,67,68,69]. On the other hand, there are only a few probiotic interventions, as shown by the meta-analysis conducted by Zawadzka et al. [70]. The authors included studies that examined the effects of probiotics, prebiotics, and synbiotics on thyroid disease in humans. However, only two articles met the inclusion criteria for further analysis, underscoring the importance of further research in this area [70].

The strain *Lactiplantibacillus plantarum 299v* (*lactobacillus plantarum 299v*, *Lp299v, Lp DSM 9843*) belongs to the *Firmucutes* type, one of the most abundant microorganisms in the human intestinal microbiota. Notably, it exhibits resistance to both low and high pH in the digestive tract, a crucial characteristic for an effective probiotic to reach the large intestine and confer benefits to the host [71]. Its pro-health activities have been increasingly widely documented in the literature in recent years [72,73]. In numerous studies, *Lp299v* has been observed to ameliorate the intensity of symptoms associated with irritable bowel syndrome (such as abdominal pain, bloating, feeling of incomplete bowel movements or normalization of bowel movements) [71,72,73,74,75,76,77]. *Lp299v* has been shown to reduce the gastrointestinal symptoms associated with enteral nutrition in cancer patients [78], including a decrease in the occurrence of flatulence [79]. However, the effect of *Lp299v* on systolic and diastolic blood pressure is ambiguous. In a six-week study in patients with stable coronary artery disease, the *Lp299v* intervention did not affect blood pressure measurements [80] or increased systolic blood pressure (*p* = 0.039) [81]. Conversely, in another six-week intervention with *Lp299v* among healthy smokers, a significant reduction in systolic blood pressure was observed (*p* < 0.001) [82]. There are also observations of the anti-inflammatory effect of the *Lp299v* strain. In two studies among patients with coronary artery disease, it reduced the concentrations of pro-inflammatory interleukins (ILs) 8 and 12; interferons, including interferon gamma (IFN-γ); and IL-1β [80,81]. *Lactobacillus plantarum 299v*, in a study in mice deficient in IL-10, relieved colitis and reduced the levels of IL-12 and IFN-γ [83]. In another study, the *Lp299v* strain increased IL-10 and decreased pro-inflammatory tumor necrosis factor-α (TNF-α) levels [80]. *Lp299v* can also benefit blood iron levels in women [84,85]. The meta-analysis by Vonderheid et al. also showed that the *Lp299v* strain, among other strains, is very effective in supporting increased iron absorption [86]. Low iron levels reduce the synthesis of thyroid hormones and thyroid peroxidase (TPO), as well as increase the risk of anti-TPO positivity, although the mechanism is not fully understood [20,87].

### Strengths and Limitations

This study has several strengths. The experiment will complement the knowledge about the impact of diet and probiotic supplementation on AIDH. Another strength is the form of education, which should be more understandable for patients, as opposed to giving dietary recommendations without explanation. In our study, we will assess whether changes in the consumption of the most important nutrients in Hashimoto’s (e.g., selenium, iodine, iron, zinc, magnesium) correlate with the change in anti-TPO, other nutritional parameters, and quality of life, which, to our knowledge, has not been investigated so far. Additionally, the effect of *Lp299v* on AIDH has not been explored, despite evidence of an immunological effect. Therefore, we will be the first to explore this area. Another strength is the length of the intervention. The mentioned studies show that 12 weeks of nutrition education brings health benefits and that only 6 weeks of *Lp299v* supplementation was enough to notice changes in inflammatory markers. It is worth emphasizing that the trial does not present any health risks to participants; the *Lactiplantibacillus plantarum 299v* used is a commercial dietary supplement (not a medical drug), and nutritional counseling aims to improve the diets of patients and is consistent with recommendations and nutritional standards.

The study limitations should also be mentioned. The first is the method of group selection, which is deliberate and snowballed. Second, the nutrient estimates from the Food Records Questionnaire are dependent on the patient’s conscientiousness and accuracy. Another limitation is that the collected data on the consumption and intake of the probiotic may be subject to error, as the researchers will rely on information provided by the patients. The authors, however, will provide detailed instructions for completing the food diary, as well as answering any questions that participants have when completing the surveys. Participants will be asked to answer the questions honestly and conscientiously. It should also be mentioned that meetings are planned every 2 weeks, which gives the opportunity to introduce changes. However, for some patients, weekly meetings are more motivating.

## Data Availability

Not applicable.
